# A Novel Integrated Multifunction Micro-Sensor for Three-Dimensional Micro-Force Measurements

**DOI:** 10.3390/s120404051

**Published:** 2012-03-27

**Authors:** Weizhong Wang, Yulong Zhao, Yafei Qin

**Affiliations:** State Key Laboratory for Manufacturing Systems Engineering, Xi'an Jiaotong University, No. 28, Xianning West Road, Xi'an, Shaanxi 710049, China; E-Mails: w.w.z.2007@stu.xjtu.edu.cn (W.W.); yafeiqin2011@163.com (Y.Q.)

**Keywords:** integrated sensor, micro-force, pressure, temperature, glass optical fiber probe

## Abstract

An integrated multifunction micro-sensor for three-dimensional micro-force precision measurement under different pressure and temperature conditions is introduced in this paper. The integrated sensor consists of three kinds of sensors: a three-dimensional micro-force sensor, an absolute pressure sensor and a temperature sensor. The integrated multifunction micro-sensor is fabricated on silicon wafers by micromachining technology. Different doping doses of boron ion, placement and structure of resistors are tested for the force sensor, pressure sensor and temperature sensor to minimize the cross interference and optimize the properties. A glass optical fiber, with a ladder structure and sharp tip etched by buffer oxide etch solution, is glued on the micro-force sensor chip as the tactile probe. Experimental results show that the minimum force that can be detected by the force sensor is 300 nN; the lateral sensitivity of the force sensor is 0.4582 mV/μN; the probe length is linearly proportional to sensitivity of the micro-force sensor in lateral; the sensitivity of the pressure sensor is 0.11 mv/KPa; the sensitivity of the temperature sensor is 5.836 × 10^−3^ KΩ/°C. Thus it is a cost-effective method to fabricate integrated multifunction micro-sensors with different measurement ranges that could be used in many fields.

## 1. Introduction

Three-dimensional (3D) micro-force measurementa are an essential part in many engineering and science applications, such as precision 3D profile measurement [1], determining of material elastic modulus and hardness of materials [2], micromanipulation of micro objects [3,4] and monitoring of insect behavior [5]. Micro-force sensors capable of meeting the needs for force measurement at the micro/nano Newton level have been studied for many years [6,7].

Various types of sensors have been developed for measuring such forces [8–14]. Muntwyler *et al.* [8] presented a three-axis capacitive force sensor that enables force measurements in a range between ±20 μN and ±200 μN with resolution down to 30 nN, but it is very difficult to fabricate and the cost is high due to the complex structure and electronic circuits for capacitance detection. Yoshikawa *et al.* [9] reported a nanomechanical membrane-type surface stress sensor (MSS) which provides highly accurate measurements. The MSS consists of an “adsorbate membrane” suspended by four piezoresistive “sensing beams” that can't be used for direct measurement of the contact force. Huang *et al.* [10] developed a touch stress sensor with four multi-layer curled cantilever beams. Fabrication of the curled micro-cantilever beams is quite complicated due to the combination of elastomer, polymer, Pt, Ti, and silicon, which is simplified in this work by only using silicon.

Among these force sensors, the piezoresistive probe sensors, fabricated on monocrystalline silicon, are the most popular type [11–14] due to their excellent performance, simple fabrication process and low cost. While these sensors still have some drawbacks like lack of an overload protection element that could be easily damaged; big measurement errors caused by temperature, pressure and electromagnetism [15] (due to the stylus probe). Sometimes, under different temperature and pressure, the characters of analytes change a lot. For example, at elevated temperatures, the mechanical properties of the polymer matrix deteriorate rapidly compared to normal temperature [16]; the elastic deformation of embryonic cell, applied by high pressure, is obviously compared to low pressure [17].

To solve these problems, this paper develops a novel integrated multifunction silicon micro-sensor. The integrated sensor chip consists of three sensors: a micro-force sensor with tunable sensitivity capability and high resolution down to sub-micro Newton levels, an absolute pressure sensor and a temperature sensor. Thereby information can be obtained at different pressures and temperatures and be more accurate.

## 2. Integrated Multifunction Micro-Sensor Design

### 2.1. Micro-Force Sensor Design

**Figure 1.**
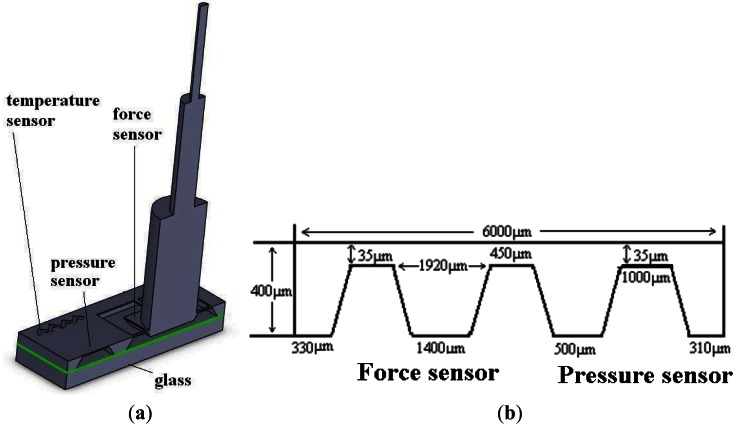
Cross-sectional view and the dimensions of the integrated sensor. (**a**) Cross-sectional view; (**b**) Dimensions.

**Figure 2.**
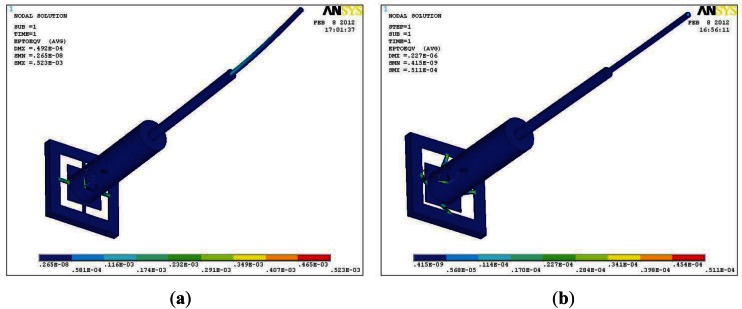
The strain distribution of the micro-force sensor. (**a**) Force (3 mN) applied in X or Y orientation; (**b**) Force (10 mN) applied in Z orientation

Figure 1 shows the structure of the integrated sensor. The micro-force sensor consists of the flexible sensing structure, the overload protection glass and the quartz optical fiber probe. Four sensing beams are used to support the central mesa in the flexible sensing structure. One glass optical fiber (GOF) probe is glued on the flexible sensing structure as the tactile probe. The glass bonded to the bottom of the sensor chip is the overload protection unit, and the central mesa-to-glass gap is 5 μm. When the sensor is overloaded, the central mesa will get in touch with the glass that could protect the cantilever beams from breaking. Force can be applied to the probe in the X, Y or Z orientations, which then leads to beam deformation as shown in Figure 2 which is simulated by ANSYS. Dimensions of the micro-force sensor are shown in Figure 1(b), the thickness of beams and pressure sensor membrane are all 35 μm. When a force is applied on the probe the membrane deforms, creating a maximum strain near both ends of the cantilever beams. Hence twelve p-type piezoresistors in the form of three Wheatstone bridges are placed on the sensing beams at the locations where the maximum strain is generated. The changes of the piezoresistors caused by strain are linearly proportional to the applied force.

For piezoresistive silicon micro-sensor, the maximum strain of resistors should be smaller than 500 micro strains [18]. In the range of strain, the sensor could achieve high linearization and sensitivity. The maximum strain, in Figure 2(a), is 523 micro-strains, so when the applied force in the X or Y orientation, is smaller than 3 mN, the micro-force sensor in the X or Y orientation could achieve high linearization and sensitivity, while in Figure 2(b), the maximum strain is only 51.1 micro-strains that could guarantee high linearization of the sensor in the Z orientation.

### 2.2. Absolute Pressure Sensor Design

**Figure 3.**
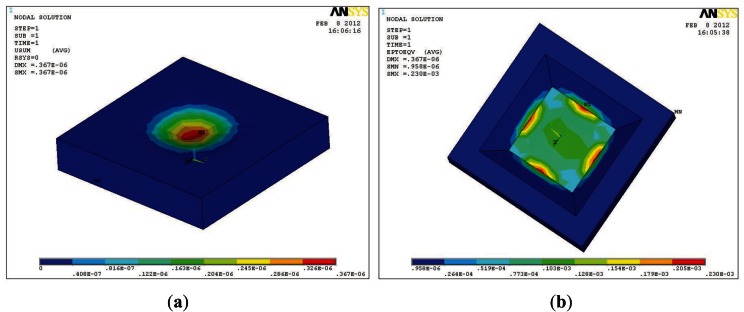
The deformation of the micro-force sensor with pressure 200 KPa applied on it. (**a**) The displacement distribution; (**b**) The strain distribution.

The absolute pressure sensor is designed as a square membrane structure. Pyrex glass is bonded to the bottom of the silicon wafer to form the vacuum chamber. When a vertical pressure is applied on the sensor, the membrane deflects vertically down, as shown in Figure 3(a). Four p-type piezoresistors are formed on the square membrane. Piezoresistors in the form of Wheatstone bridges are placed near frame of the membrane where maximum strain is generated, as shown in Figure 3(b). Due to the strain in the membrane the resistivity of the p-diffused piezoresistor begins to change. The mechanical signal can be converted into electrical signals by using the imbalance of the excited Wheatstone bridge circuits. To achieve good linearity, the biggest deflection of the membrane must be smaller than a fifth of the membrane thickness and the maximum strain of the resistors should be smaller than 500 micro strains. Due to the sensor measurement range is 0–200 KPa, the maximum deflection and strain of the membrane, 200 KPa pressure applied on the sensor, are calculated by the software ANSYS and shown in Figure 3. It is proved that the maximum deflection is only 0.364 μm, shown in Figure 3(a), which is much smaller than fifth of membrane thickness (35 μm, shown in Figure 1(b)). The maximum strain is only 229 micro strains, shown in Figure 3(b), which is much smaller than 500 micro strains, so we conclude that the absolute pressure sensor can achieve high linearization.

### 2.3. Resistors and Temperature Sensor Design

The thermistors of the temperature sensor and the piezoresistors of force sensor and pressure sensor, formed by ion implanting, are all sensitive to the temperature and pressure simultaneously. To guarantee the accuracy of these sensors, we have to make huge efforts to minimize the cross interference.

There two kinds of silicon resistors, p-type silicon resistor (p-Si formed on the n-type silicon wafer by boron ion doping) and n-type silicon resistor (n-Si formed on the P-type silicon wafer by phosphorus ion doping). As shown in Table 1 [19], p-Si in [11̄0], [110] crystal orientation and n-Si in [100], [010] crystal orientation are both suitable for accelerometer and pressure sensors with their their high piezoresistance coefficient, while only p-Si in [100], [010] crystal orientation is suitable for temperature sensoras due to its low piezoresistive coefficient, so the (100) n-type SOI wafer is selected to fabricate the multi-sensor chip to form p-type silicon resistors.

**Figure 4.**
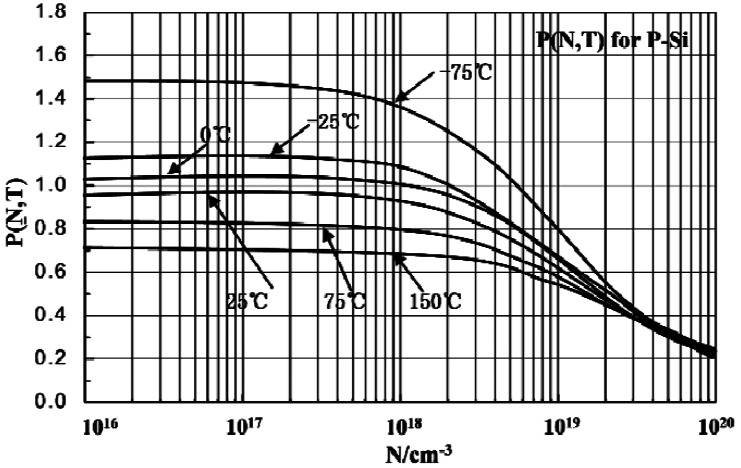
The piezoresistance coefficient *versus* boron ion doping dose and temperature.

In the range of BC (−55 °C–175 °C), the resistivity of p-type silicon resistors increases linearly proportional to the temperature [20]. Figure 4 shows the piezoresistance coefficient (denoted as P) of the resistors according to different doping concentration (denoted as N) and temperature (denoted as T) [21]. Depending on the temperature increasing, the piezoresistance coefficient decreases, in the range of 0–3 × 10^19^ cm^−3^ boron ion doping concentration. At some stable temperature in the range of −75 °C –150 °C, when the boron ion doping concentration is more than 3 × 10^17^ cm^−3^, the piezoresistance coefficient decreases gradually.

**Figure 5.**
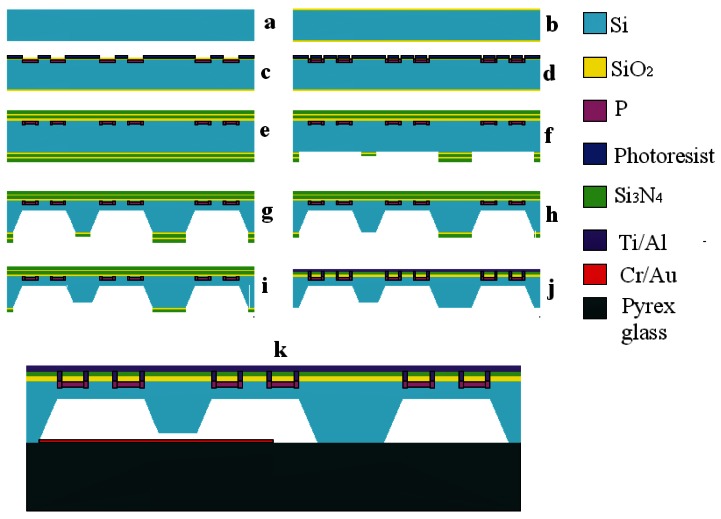
The process of fabricating the micro-sensor chip.

Sensitivity of the force sensor and pressure sensor can be adjusted by varying the boron ion doping concentration. Lower boron ion doping concentration gives a higher piezoresistive coefficient and higher sensitivity, but also higher temperature interference, while in the range of 3 × 10^18^ cm^−3^ and 5 × 10^18^ cm^−3^ boron ion doping concentration, the temperature interference for resistor and piezoresistive coefficient can mutual offset, so to get high sensitivity and low temperature interference, 5 × 10^18^ cm^−3^ is selected as suitable boron ion doping concentration to form force sensor and pressure sensor resistors. Some methods are employed to minimize interference of the temperature sensor resulting from the applied pressure: high boron ion doping concentration 2 × 10^20^ cm^−3^, with low piezoresitive coefficient (shown in Figure 5), is selected to form the thermistors; the thermistors are located at the surface of silicon wafer above the silicon-glass bonding area where the strain resulting from the applied pressure is small; the thermistors are designed as wave type structures arranged along the [100] and [010] crystal directions, shown in Figure 1, which achieves the minimum piezoresistive coefficient of the (100) silicon.

## 3. Fabrication of Integrated Multifunction Micro-Sensor

### 3.1. Fabrication of Micro Sensor Chip

**Figure 6.**
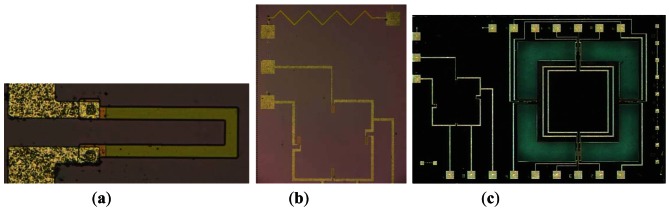
The digital microscopy of finished sensor chip: (**a**) the resistor of the pressure sensor; (**b**) the temperature sensor and the absolute pressure sensor; (**c**) the integrated sensor chip.

**Figure 7.**
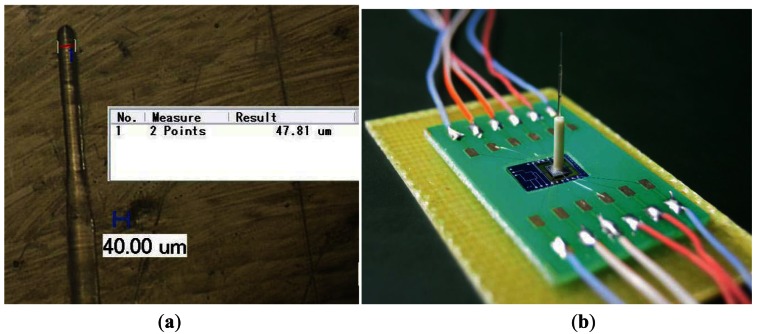
The pictures of the finished sensor. (**a**) The digital microscopy of finished probe tip; (**b**) The digital photograph of the finished sensor.

**Figure 8.**
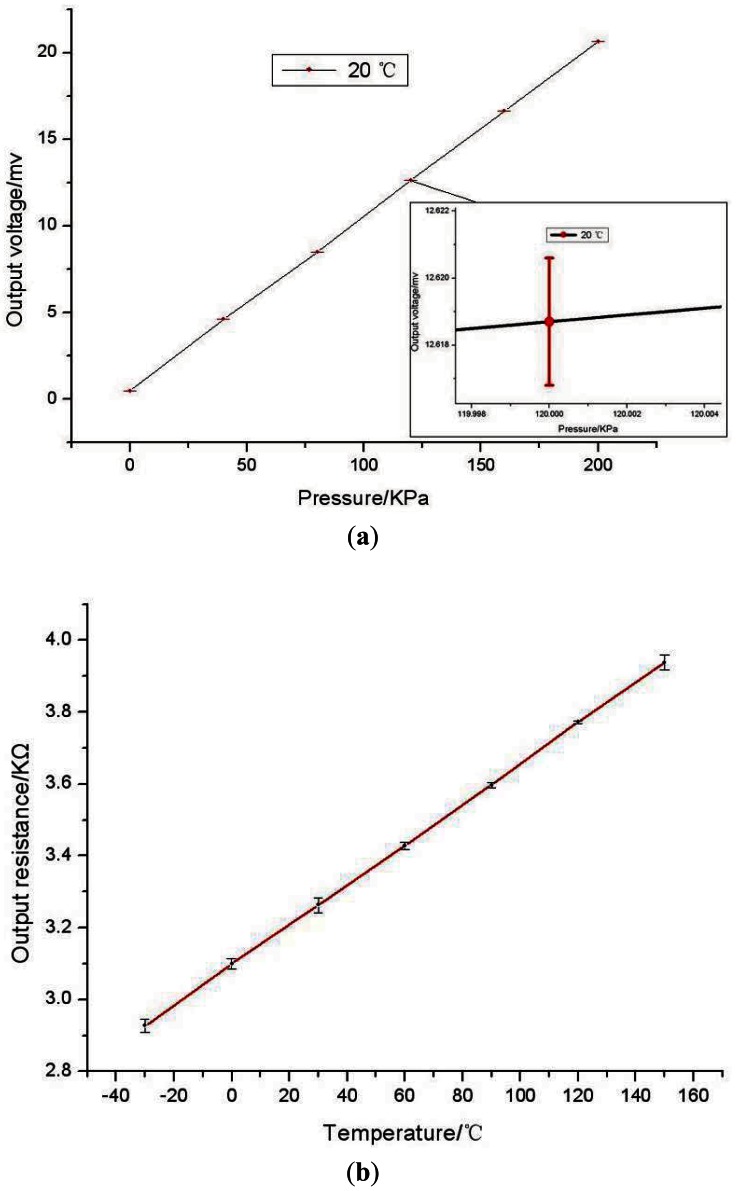
The characteristic curves of the sensors. (**a**) The pressure sensor; (**b**) The temperature sensor.

**Figure 9.**
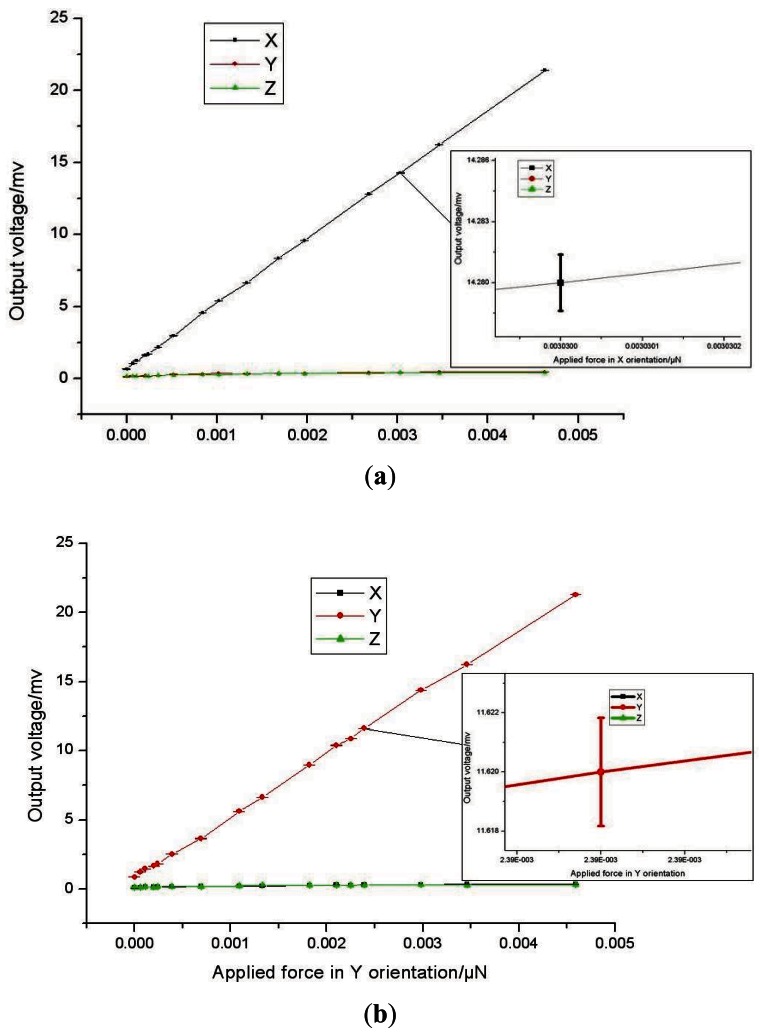

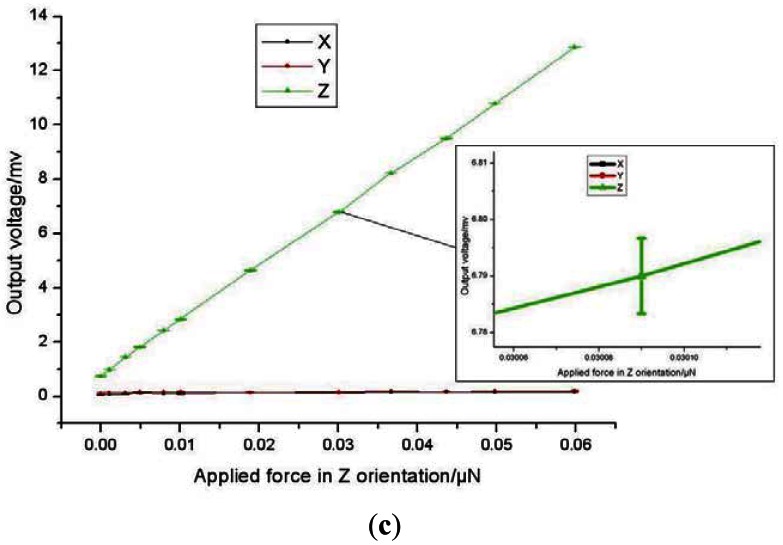
The output voltage of the micro-force sensor *versus* applied force. (**a**) Applied force in X orientation; (**b**) Applied force in Y orientation; (**c**) Applied force in Zorientation.

Silicon micromachining technology is used to fabricate the sensor chip. The process starts with a double-side-polished four-inch N-type (100) oriented single-crystal Si wafer (Figure 6(a)). The fabrication steps are as follows (Figure 5): (1) Grow an oxide (SiO_2_) layer on the surface of the Si wafer by thermal oxidation in a heating oven (Figure 5(b)); (2) Pattern the piezoresistors and piezoresistors contact area to the oxide surface by photolithography (Suss MA6), then form them by boron ion doping in an ion implantation system (Figure 5(c,d)); (3) two layers of SiO_2_/silicon nitride(Si_3_N_4_) bi-material film, which can reduce the effect of the thin film stress in the process of film fabrication, are deposited by low-pressure chemical vapor deposition (LPCVD ASM LB35) to be used as two layer masks for wet anisotropic etching on the backside of wafer and to protect the resistors on the front-side of wafer from being corroded (Figure 5(e)); (4) Pattern the back structure of the integrated sensor to the bi-material film surface by photolithography, and remove the Si_3_N_4_ layer by phosphoric acid (H_3_PO_4_) solution, remove the SiO_2_ layer by buffered hydrofluoric acid (BHF) solution (Figure 5(f)); (5) The backside of the integrated sensor chip is etched in KOH solution to about 365 μm (Figure 5(g)); (6) Pattern the bottom of the central mesa to the bi-material film surface by photolithography, remove the last SiO_2_/Si_3_N_4_ layer (Figure 5(h)); (7) The backside of the silicon wafer is immersed in KOH solution again to form the narrow gap (5 μm) between the bottom of central mesa and Pyrex glass, complete the final formation of the backside of the integrated sensor (Figure 5(i)); (8) Pattern the contact hole to the bi-material film surface of the silicon wafer front side by photolithography, and remove the SiO_2_/Si_3_N_4_ by reactive ion etching (RIE) (Figure 5(j)); (9) Ti/Al bi-layer film is deposited by physical vapor deposition (PVD, Varian 3180 ion sputtering coator) (Figure 5(j)), and then the metal wire is etched by RIE, Figure 6(c) shows the distribution of the metal wire; (10) The cantilever beams of the force sensor are formed by Inductively Coupled Plasma (ICP, STS ICP ASE) etching; (11) Cr/Au bi-layer is deposited on the Pyrex glass, as the metal electrode, to avoid the electrostatic adhesion between the central mesa and Pyrex glass (Figure 5(k)); (12) The silicon and glass is bonded by the anodic bonding technology (Suss SB6 VAC Substrate Bonder, Figure 5(k)); (13) Residual stress of the fabrication process is released by annealing. The finished sensor chip is shown in Figure 6.

### 3.2. Fabrication of Micro-Force Probe

As the tactile element of the micro-force sensor, the probe translates the applied force to the sensor chip. It's very critical to design and fabricate the probe to obtain high sensor sensitivity and accurate measurements. The probe is designed as a ladder structure to get a sharp tip and broader base. With the sharp tip, the micro-force sensor can measure the applied force more accurately. For example, when the probe tip scans over a specimen, which has a small hole on its surface, a sharp tip will sense this hole, while a blunt tip will not [22]. The broader base of the probe means a larger contact area, which is beneficial to keep the probe more securely glued on sensor chip and make the measurement range be larger in the X and Y orientation. Also with the ladder structure, the dead weight of the probe should be lighter and output voltage of the force sensor with no force applied should be smaller.

Over decades, GOF has been used in many fields for measuring physical properties or operating objects. GOF is selected as the probe because of its light weight, immunity to electromagnetic interference (EMI), small size, high sensitivity, and capability of operating at high temperatures [17]. As GOF are made with 99% of silica glass, the Young modulus of the probe is close to that of silicon chip, specifically, the former is 73 GPa (for silica), while the latter is 130 Gpa [for silicon in (100) crystal face]. The coefficient of thermal expansion of silica is only 0.55 × 10^−6^/°C, which can minimize the temperature effects when the sensor works under different temperatures.

The GOF has a cladding around 125 μm in diameter that can be etched by buffer oxide etch (BOE) solution, which contains a mixture of HF and NH4F. The GOF tip is dipped into the BOE solution filled in a plastic container in the water bath at 40 °C. The finished probe tip is shown in Figure 7(a). The sharp tip is only 47.81 μm in diameter.

### 3.3. Package of the Multifunction Micro-Sensor

Once the desired diameter of the fiber is obtained after etching, the next step is to properly glue the etched fiber on the micro-force sensor chip and fixed the integrated sensor chip on the PCB to finish the package of the multifunction micro-sensor.

The transparent organic glass shell which is a piece of acrylic, with a hole on it, is used for probe position and fixed. The packaging steps are as follows: first, place the sensor chip in the blind hole of the PCB and glue them together by epoxy resin adhesive, solidification occurs at 60 °C for two hours; then, drop a little epoxy resin adhesive on the central mesa of the micro-force sensor chip and place the transparent organic glass over the PCB, the probe is fixed on the central mesa through the hole of the transparent organic glass; at last, after solidification, the shell is taken off from the PCB and down-lead the micro-sensor chip by gold wire bonder, the finished sensor is shown in Figure 7(b).

## 4. Results and Discussion

The characterization of absolute pressure sensor is measured by a precision manometer with an accuracy class of 0.02 at 20 °C and is repeated six times. Figure 8(a) shows the relationship between the average output voltage and applied pressure, with 5 V DC voltage powered. In the range of 0–200 KPa, the measured sensitivity of the pressure sensor is 0.11 mv/KPa, non-linearity is 0.37% FS, the offset of the pressure sensor is 0.533 mv and the precision of measurements is 0.425% FS.

The error bar is mean square of the output voltage, it is caused by zero drift and the assembly pre-tightening force. The experimental results are well matched to the simulation results, in the range of 0–200 KPa, the absolute pressure sensor can achieve high linearization.

The temperature sensor is measured in a temperature controller in the range of −30 °C–150 °C and experiment process is repeated for six times. The response of the temperature sensor is shown in Figure 8(b). The sensitivity of the temperature sensor is 5.836 × 10^−3^ KΩ/°C, non-linearity is 0.44% FS (less than 175 °C). The temperature coefficient of resistance (TCR) of the temperature sensor is determined to 1.82 × 10^−3^/°C. The error bar is mean square of the output piezoresistance, it is mainly caused by self-heating of the piezoresistors.

A micro-force measurement system is built up consisting of a precision positioning micro stage with three degrees of freedom and resolution up to nanometre scale, an analytical balance with resolution up to 0.01 mg (about 100 nN). When the sensor probe is loaded on the analytical balance, we can read out the applied force and corresponding output voltage. In this process, a series of forces are applied to the sensor, which produces the corresponding output signals. Then, correction lines between the output signal of the sensor and the actual force can be obtained, shown in Figure 9 when the sensor is equipped with 25 mm length probe. In these figures, the correction lines do not go through the origin and there exists a voltage output with no force applied. The phenomenon is caused by the dead weight of the probe and fabrication error of the resistors. The boron ion doping in the silicon is not distributed very uniformity and this leads to differences between the resistors. The error bar indicates the instability of the force sensor. When the force is applied on the sensor the output voltage is not a very stable value, and 10 voltage values were collected *per* applied force.

**Figure 10.**
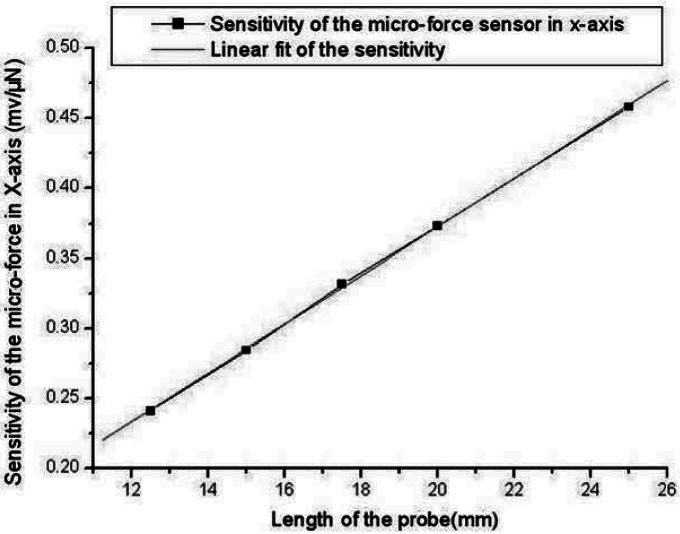
The sensitivity of the micro-force sensor in X-axis *versus* the length of the probe.

The sensitivity, resolution, cross-term interference, non-linearity and precision of measurements of the micro-force sensor separately is 0.4582 mV/μN, 300 nN, 2%FS, 0.46% FS and 4.98% FS in the X orientation; 0.4570 mV/μN, 400 nN, 1.5% FS, 0.46% FS and 5.16% FS in the Y orientation; 0.0205 mV/μN, 12 μN, 1.63% FS, 0.5% FS and 3.68% FS in the Z orientation. The lateral sensitivity of the force sensor is much bigger than that of the sensor developed by Tibrewala *et al.* (11.29 mV/V/mN) [10]. The minimum force that can be detected is 300 nN that is obviously superior to the sensor developed by Tibrewala *et al.* (9 μN) [10]. Performances of the micro-force sensor in the X orientation and Y orientation are almost the same, resulting from the symmetrical structure. As the applied force has been amplified by the probe which works as a lever, the sensitivity and resolution of the X and Y orientation are obviously higher than the Z orientation. Different sensitivity of the force sensor in the X and Y orientation can be obtained through variation of the lever length. Five probes of different lengths were fixed on sensor chips. Experimental results, shown in Table 2 and Figure 10, illustrate that the sensitivity of micro-force sensor in the x-axis is linearly proportional to the probe length and can be indicated as follow:

(1) S=0.01739L+0.0248

**Figure 11.**
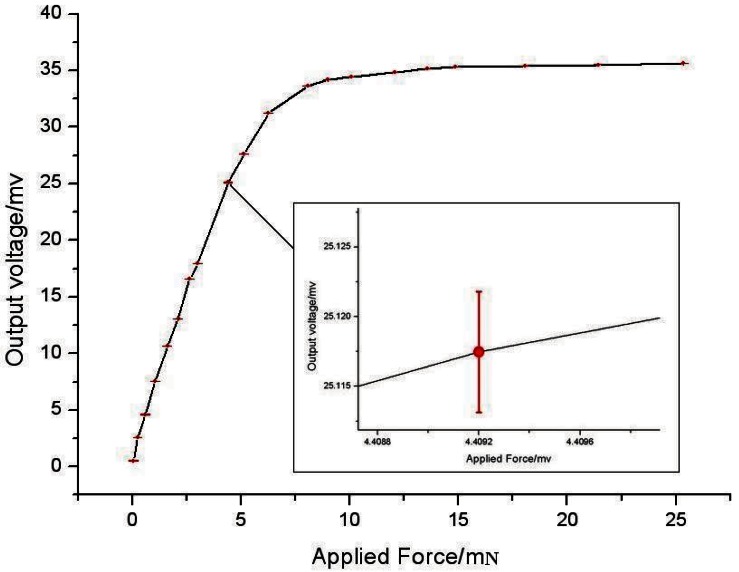
The overload curves of the sensor in Z orientation.

The non-linearity is 0.54% FS resulting from the sensor package and sensor chip differences. The overload prevention function of the sensor in Z orientation has also been checked. The results, shown in Figure 11, indicate that in the range of 0–5 mN the force sensor in Z orientation could achieve high linearization, and when the applied force is more than 10 mN the sensor would be protected.

## 6. Conclusions

An integrated multifunction micro-sensor for precision micro-force measurements has been developed. By choosing an appropriate boron ion doping dose, the output of the temperature sensor can maintain good linearity over the whole temperature range, and the force sensor and pressure sensor can maintain high sensitivity and low temperature effectd. The location and the crystal orientation arrangement of the temperature sensor minimize the effects of stress. The resolution of the tri-axis micro-force is down to 300 nN. Micro-force sensors with different sensitivity and resolution can be obtained by varying the probe length. The integrated sensor can be used in many engineering and science applications, especially for precision measurement of object profiles and mechanical properties under different pressure and temperature conditions.

## Figures and Tables

**Table 1.** The piezoresistance coefficient of p-Si and n-Si.

**Longitudinal crystal orientation**	**Longitudinal piezoresistance coefficient πl**	**Transverse crystal orientation**	**Transverse piezoresistance coefficient πt**	
[100]	6.6	[010]	−1.1	p-Si
	−102.2		53.4	n-Si
[010]	6.6	[001]	−1.1	p-Si
	−102.2		53.4	n-Si
[11̄0]	71.8	[110]	−66.3	p-Si
	−31.2		17.6	n-Si
[110]	71.8	[11̄0]	−66.3	p-Si
	−31.2		17.6	n-Si

**Table 2.** Relationship between the probe length and sensitivity.

Length of the probe(mm)	12.5	15	17.5	20	25
Sensitivity in X-axis(mv/μN)	0.2411	0.2846	0.3316	0.3732	0.4582
